# Electrical burn-induced vocal cord injury: insights from a case report and literature review

**DOI:** 10.1080/23320885.2024.2374549

**Published:** 2024-07-10

**Authors:** José Fonseca, Jorge Garza, Mauricio García, Karen Aguirre, Haya Alotaibi, Hatan Mortada

**Affiliations:** a Plastic Surgery Department, Hospital Universitario “Dr José Eleuterio González”, Universidad Autónoma de Nuevo León, Monterrey, Nuevo León, México; b General Surgery department, centro médico nacional del noreste UMAE#25 del Instituto Mexicano del Seguro social, Monterrey, Nuevo León, México; c Department of Surgery, College of Medicine, Princess Nourah bint Abdulrahman University, Riyadh, Saudi Arabia; d Division of Plastic Surgery, Department of Surgery, King Abdullah Bin Abdullaziz University Hospital, Riyadh, Saudi Arabia; e Division of Plastic Surgery, Department of Surgery, King Saud University Medical City, King Saud University, Riyadh, Saudi Arabia; f Department of Plastic Surgery & Burn Unit, King Saud Medical City, Riyadh, Saudi Arabia

**Keywords:** Electrical injuries, vocal cord complications, high voltage, electrical burn injury, burn

## Abstract

We report a rare case of vocal cord injury from an electrical burn, managed successfully with conservative, non-invasive treatment. This unique case illustrates potential complications of electrical trauma and underscores the need for vigilance and consideration of conservative management approaches.

## Introduction

Electrical injuries can occur due to various causes such as lightning strikes, and exposure to low or high voltage currents [1]. These injuries frequently carry a high risk of morbidity and mortality [2]. It is important to note that almost all electrical injuries are accidental and, importantly, often preventable. In cases where they are not immediately fatal, electrical injuries can cause extensive damage, potentially leading to the dysfunction of multiple tissues or organs [1–4]. This broad range of impact underscores the critical need for effective safety measures and immediate medical response to minimize the long-term consequences of such injuries. The primary aim of this case report is to highlight a rare and underreported consequence of electrical injuries, specifically focusing on vocal cord injury resulting from an electrical burn. Through detailed documentation and analysis of this case, we seek to contribute to the existing body of knowledge on the varied and complex presentations of electrical injuries. Additionally, this report aims to underscore the importance of considering and recognizing less common complications in patients who have suffered electrical burns, thereby aiding in prompt and accurate diagnosis and treatment.

## Case study

A 38-year-old male patient, with no significant medical history, suffered an electric shock from a street wire with a voltage of 13,800 volts. The electric shock involved a left hand entry point and a right retroauricular region exit point. This incident occurred while the patient was cleaning the roof of his house. The patient was reportedly in contact with an exposed electrical source, which resulted in the shock. Upon initial assessment, he exhibited symptoms consistent with electrical injury but no immediate life-threatening conditions.

Upon admission to the emergency department, the patient was evaluated with a Glasgow Coma Scale score of 15, indicating full consciousness. His vital signs showed normotensive and tachycardic, with an oxygen saturation of 98% on room air (ambient FiO2). The physical examination revealed that he had sustained both superficial and deep second-degree burns on his face, the right retroauricular region, and thorax. Additionally, he had more severe third-degree burns on the left upper limb. These burns collectively covered approximately 25% of his total body surface area (TBSA). Upon arrival to the emergency room, there were no signs of inhalation injury such as soot in the mouth or trachea, ignited clothing, or burnt facial hair. In the intubation note from the anesthesiologist, who performed the orotracheal intubation under direct vision, the finding is referred to as airway edema, airway burn, carbonaceous sputum, and describes a bloody lesion on the affected vocal cord. Due to lack of equipment, initial video laryngoscopy could not be performed upon arrival to the emergency room.

**Figure 1.**
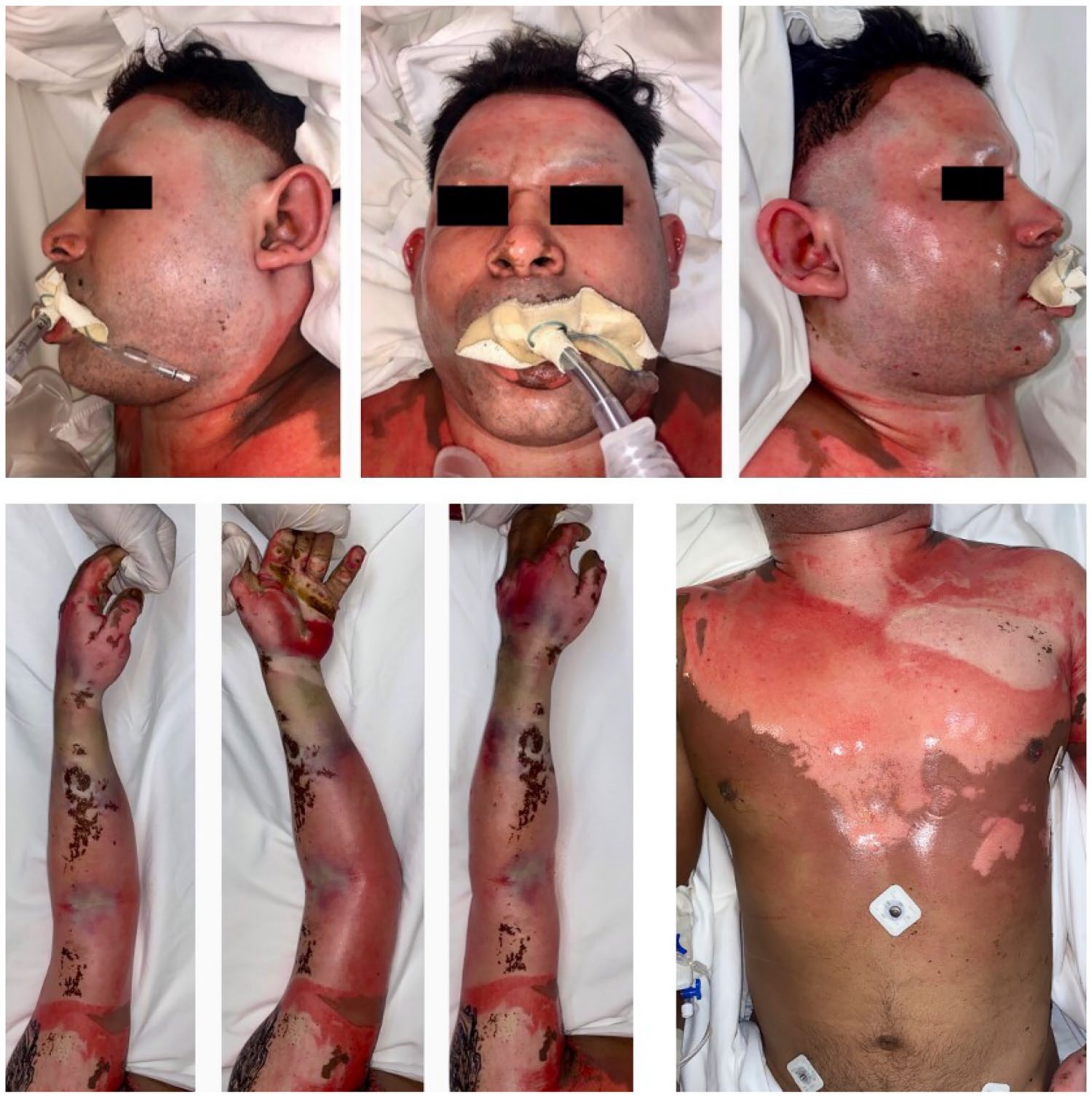
Appearance of the patient early after admission to the intensive care unit.

**Figure 2.**
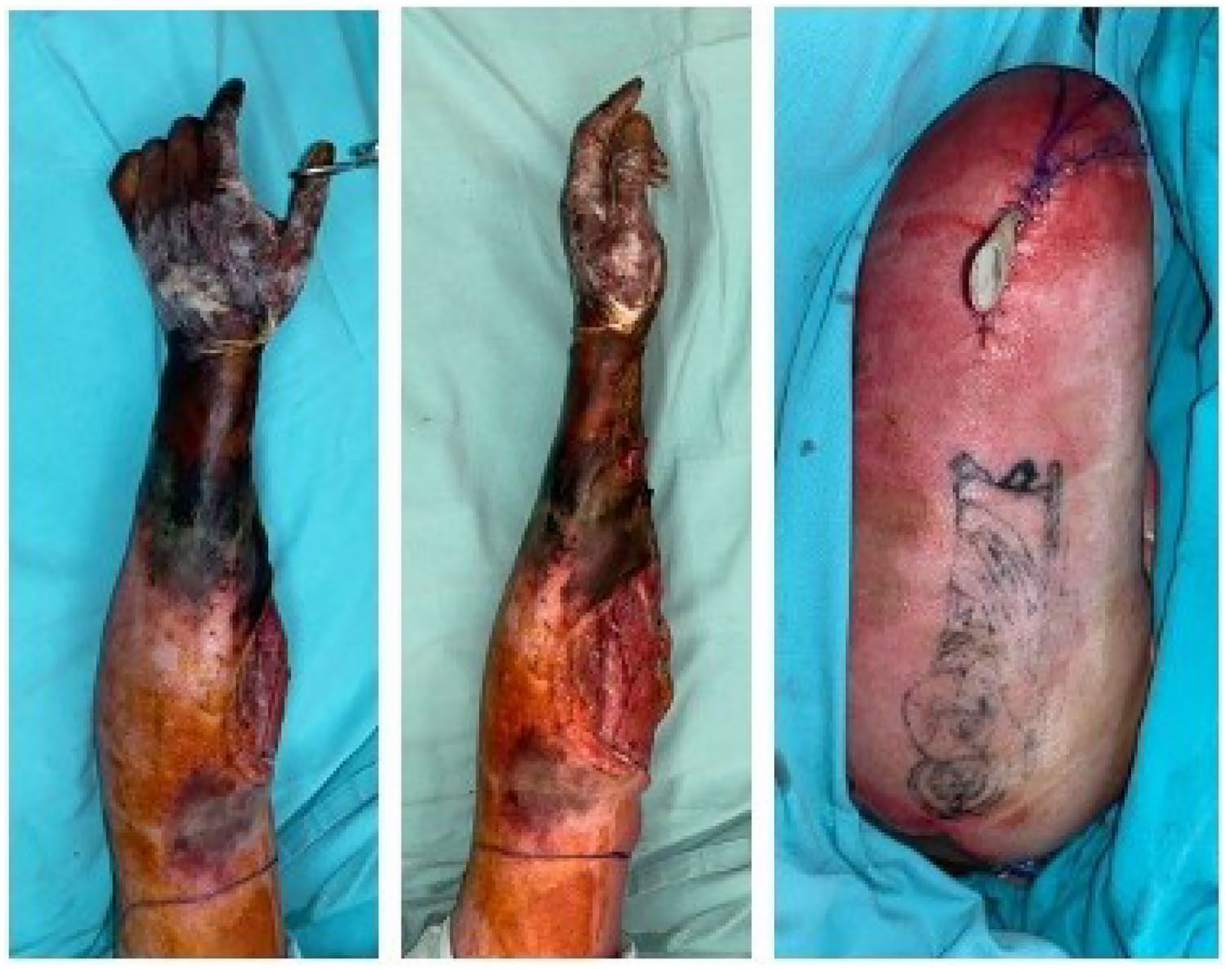
Left upper limb dry gangrene, as depicted in the first two pictures, with the last picture showing the limb after amputation.

A notable clinical finding was the presence of stridor, a sign of potential airway compromise, which prompted the healthcare team to perform assisted mechanical intubation. This intervention was crucial for maintaining the patient’s airway and ensuring adequate ventilation. Furthermore, the patient exhibited signs of compartment syndrome in his left forearm, a serious condition resulting from increased pressure within the muscle compartments. Consequently, a fasciotomy was performed as an emergency procedure to relieve this pressure and prevent further tissue damage (as depicted in Figure 1). During his hospitalization, the patient underwent a series of significant medical interventions. Notably, amputation of the left upper limb was necessitated due to severe vascular compromise (Figure 2). This drastic measure highlights the extent of the damage caused by the electrical injury. Additionally, an autologous skin transplant was performed, aiding significantly in his recovery process.

**Figure 3.**
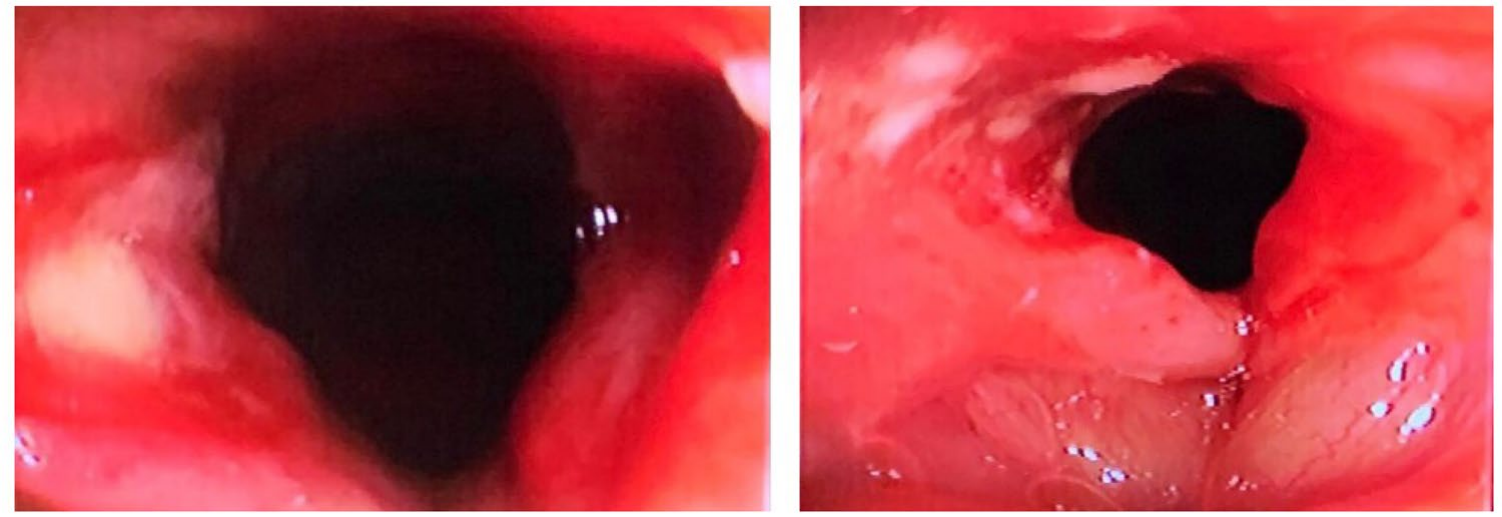
Images obtained from bronchoscopy represent the vocal cords, showing the left vocal cord without anatomical compromise and the right vocal cord with the presence of electrical burn lesions.

This positive development led the medical team to initiate the process of weaning him off assisted mechanical ventilation. Successfully transitioning from mechanical support is a crucial step in the recovery from severe injuries and respiratory compromise. Subsequent to the extubation, a control bronchoscopy was conducted to assess the airways. This examination revealed a lesion on the right vocal cord, which was identified as a secondary complication of the path of the electrical burn (Figure 3).

The patient’s postoperative course involved continued conservative medical treatment, which proved to be effective. Remarkably, he remained asymptomatic throughout this period, exhibiting no signs of airway compromise, dysphagia (difficulty swallowing), or dysphonia (voice disorder). This absence of complications is noteworthy, considering the severity and extent of the initial injuries, including the lesion on the vocal cord. The patient was intubated for 10 days in intensive care. Although the literature states that prolonged intubation (greater than 14 days) increases the risk of vocal cord injury, the complications described as a result of this are generally complications in both vocal cords, most of the time. We do not know with certainty if the injury was caused by the intubation or if it was aggravated by it, but the findings in the anesthesiologist’s intubation note give us a high index of suspicion that the injury was caused by the electric burn

**Figure 4.**
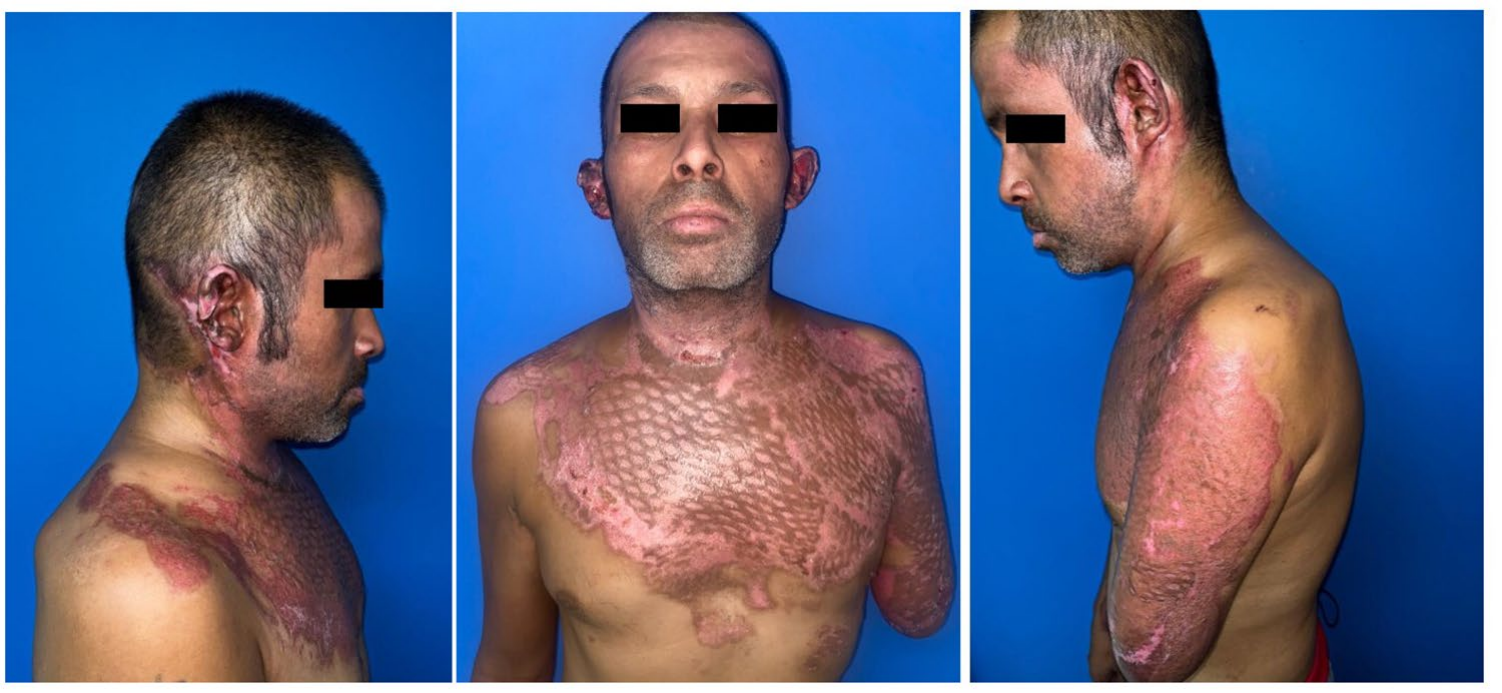
Three months after hospital discharge, the appearance of the patient showed all grafts satisfactorily integrated.

After a 34-day hospital stay, which included comprehensive care and monitoring, the patient’s condition improved significantly. The successful management of his complex injuries and the absence of any major postoperative complications allowed for his eventual discharge. At the time of discharge, the patient’s overall health had significantly improved, allowing him to leave the hospital, as depicted in Figure 4. However, it’s important to note that some degree of dysphonia persisted. Due to financial constraints, the patient was only able to attend one follow-up consultation, during which persistent dysphonia was noted. Unfortunately, we were unable to conduct a comprehensive ENT examination to fully assess the patient’s vocal cord function and determine the extent of recovery from the initial injury. The patient was subsequently lost to follow-up, limiting our ability to document long-term outcomes. Informed consent was obtained from the patient for the publication of this case report, ensuring adherence to ethical standards while maintaining confidentiality and anonymity.

## Discussion

Electrical injuries are a complex form of trauma often associated with high morbidity and mortality. The severity of the injury depends on the type of current, voltage, and resistance [1,2]. In this case report, we presented the challenging management of a 38-year-old male who suffered severe electrical injuries, including a unique vocal cord lesion and extensive burns, leading to interventions such as mechanical ventilation, fasciotomy, and left upper limb amputation, and concluding with a successful recovery after a 34-day hospitalization.

There are four main types of electrical injuries: flash, flame, lightning, and true electrical injuries. Flash injuries, caused by an electric arc, are generally associated with superficial burns, as no electric current passes through the skin [1,3]. Flame injuries occur when an electric arc ignites a person’s clothing; in these cases, the electric current may or may not pass through the skin [2–4]. Lightning injuries, which involve extremely short but very high voltage electrical energy, are associated with an electrical current flowing through the individual’s entire body. True electrical injuries involve an individual becoming part of an electrical circuit, where entry and exit sites are usually found [1–4].

Of all burns treated in medical settings, 4% to 5% are electrical in nature. In the United States, accidental high-voltage electrical injuries result in approximately 400 deaths annually [5]. Among adults, electrical injuries are most commonly associated with occupational hazards; conversely, in children, domestic electrical incidents are more prevalent [5,6]. Men are more frequently injured by electricity compared to women. The hands are the most common initial contact point for these injuries, followed by the head, while the feet often serve as the grounding point [5–7]. Burns can be classified as high or low voltage. High voltages, typically above 500-1000 volts, cause deep burns and extensive damage to deep tissues and organs. In contrast, low voltage exposures generally result in minor injuries [8–11]. Complications of electrical injuries share similarities with those of other thermal burns, such as the risk of infection, which can progress to sepsis, compartment syndrome, and rhabdomyolysis, the latter resulting from extensive muscle damage due to internal burns [10]. Additionally, electrical shocks can cause associated injuries, such as those sustained from being thrown from the electrical source or falling from a height (e.g. roofs, trucks, ladders) [11]. These injuries, which may include long bone fractures, spinal fractures, lacerations, and pneumothorax, should be thoroughly evaluated and treated appropriately [10–12].

Cardiac complications are a possibility following electrical injuries [11]. In addition to causing arrhythmias and other electrical disturbances, electrical injury can directly damage cardiac myocytes. Consequently, patients may experience late arrhythmias due to this damage, such as sinus tachycardia or premature ventricular contractions [12,13]. However, electrical injuries that result in long-term cardiac sequelae are uncommon [11–13].

If the electrical current’s pathway through the body crosses the thorax, there is a risk of paralysis of the chest wall muscles, leading to potential respiratory arrest [12]. Nonetheless, unlike cardiac myocytes, lung tissue is a poor conductor of electricity and is, therefore, less likely to suffer direct electrical injury [12–14]. Our case report represents the third documented case in the literature concerning vocal cord injury following an electrical burn. The first case, reported by Nicole Kloosterman et al. in OTO Open 2021, detailed a 19-year-old man who suffered bilateral vocal fold paralysis following an electrical injury involving a homemade wood-carving device. The patient experienced significant respiratory distress and was managed with tracheostomy, but unfortunately showed no improvement in vocal fold motion over time [15].

The second case, presented by Akash Varshney et al. and published in 2023, involved a young male who developed unilateral vocal cord palsy following an electric injury on his arm. This case was notable for its multidisciplinary approach to management, including steroids and speech therapy, which led to an improvement in his condition [16].

In contrast to these two cases, our case report adds new insights into the spectrum of vocal cord injuries due to electrical burns, emphasizing the varied presentations and outcomes of these rare but serious incidents. This addition to the literature underscores the need for heightened awareness and prompt, comprehensive management strategies for such injuries. The mechanism of injury to the vocal cords in our patient can be explained by the physics of electrical current and the path of least resistance. In electrical injuries, the current tends to travel through the body along the path that offers the least resistance, which includes blood vessels, nerves, and other conductive tissues [17]. Given the entry point at the left hand and the exit point at the right retroauricular region in our patient, the current likely traversed the neck and laryngeal region, potentially affecting the vocal cords due to their anatomical proximity to the current’s path [18]. The rarity of vocal cord injury in electrical burn cases raises important questions about the timing of diagnosis and the role of early evaluation. In our case, the anesthesiologist’s intubation note described a bloody lesion on the affected vocal cord, which was observed immediately upon the patient’s arrival to the emergency room. This finding suggests that a thorough examination of the airway and vocal cords during the initial assessment might have revealed signs of injury. However, the priority in the acute setting is often to secure the airway and manage life-threatening conditions, which may overshadow the diagnosis of vocal cord injury. In cases where the neck is involved in the pathway of the electric current, we propose that vocal cord injury should be actively evaluated during the initial examination. By incorporating a targeted examination of the vocal cords in the initial assessment of electrical burn patients with neck involvement, healthcare providers may be able to identify and address vocal cord injuries more promptly, potentially leading to earlier intervention and improved outcomes.

One limitation of our case report is the lack of a comprehensive ENT examination to assess the patient’s vocal cord function and determine the extent of the injury. Due to financial constraints, the patient was only able to attend one follow-up consultation, during which persistent dysphonia was noted. Future case reports should prioritize comprehensive follow-up to better characterize the long-term outcomes of these rare but potentially debilitating complications.

## Conclusion

Electrical burns are capable of causing a wide array of lesions across all body organs, some of which may not be immediately apparent. Through our comprehensive review of the literature, we found no reports of cases identical to ours, wherein a patient suffered vocal cord injury as a secondary complication of electrical burns. This absence of similar documented cases underscores the rarity and uniqueness of our patient’s condition. Given the lack of previous literature on this specific complication, our case report holds significant importance in contributing new knowledge to the medical community. It emphasizes the need for healthcare professionals to be vigilant in considering such potential complications when evaluating patients with electrical burns. This report not only adds to the existing body of knowledge but also serves as a crucial reminder of the unpredictable and extensive nature of injuries caused by electrical burns.
